# Associations between chrono-nutrition and glucose metabolism across levels of glucose impairment: The Maastricht Study

**DOI:** 10.1007/s00394-026-03964-2

**Published:** 2026-04-21

**Authors:** Marvin Y. Chong, Annemarie Koster, Bastiaan E. de Galan, Carla J. H. van der Kallen, Marleen M. J. van Greevenbroek, Martijn J. L. Bours, Matty P. Weijenberg, Simone J. P. M. Eussen

**Affiliations:** 1 Department of Epidemiology, CARIM Cardiovascular Research Institute Maastricht, Maastricht University, Maastricht, The Netherlands; 2 Department of Epidemiology, GROW Research Institute for Oncology and Reproduction, Maastricht University, Maastricht, The Netherlands; 3 Department of Epidemiology, CAPHRI Care and Public Health Research Institute, Maastricht University, Maastricht, The Netherlands; 4 Department of Social Medicine, CAPHRI Care and Public Health Research Institute, Maastricht University, Maastricht, The Netherlands; 5 Department of Internal Medicine, Maastricht University Medical Center+ (MUMC+), Maastricht, The Netherlands; 6 Cardiovascular Research Institute Maastricht (CARIM), Maastricht University, Maastricht, The Netherlands; 7 Department of Internal Medicine, Radboud University Medical Centre, Nijmegen, The Netherlands

**Keywords:** Chrono-nutrition, Fasting plasma glucose, HbA1c, Normal glucose metabolism, Prediabetes, Type 2 diabetes

## Abstract

**Purpose:**

Chrono-nutrition, defined as the timing, frequency, and regularity of food intake, may influence glucose metabolism and the risk of type 2 diabetes mellitus (T2DM) through its interaction with circadian rhythms. We examined cross-sectional associations of meal frequency, irregularity, and length of the eating window with glucose metabolism in individuals with normal glucose metabolism, prediabetes, and T2DM.

**Methods:**

Data were derived from 3,467 participants of a population-based cohort enriched with T2DM. Glucose metabolism status (normal, prediabetes, T2DM) was determined by oral glucose tolerance test and use of glucose-lowering medication. Fasting plasma glucose, 2 h post-load glucose, and HbA1c were measured in venous plasma. Chrono-nutrition was assessed by questionnaire. Multinomial logistic regression estimated odds ratios for prediabetes and T2DM versus normal glucose metabolism. Linear regression models assessed associations with glycemic markers, stratified by glucose metabolism status. Analyses were adjusted for sociodemographic, lifestyle, and clinical factors, including diet quality and caloric intake.

**Results:**

A longer eating window was associated with higher odds of prediabetes (OR per hour: 1.07, 95% CI 1.01–1.13). In normal glucose metabolism (N = 2264), breakfast skipping showed higher fasting glucose (β: 0.09 mmol/L, 0.04–0.14), while in participants with prediabetes (N = 604) breakfast skipping was associated with lower 2 h post-load glucose (β: −0.50 mmol/L, −0.96, −0.05). In prediabetes, higher meal irregularity was associated with higher fasting glucose (β: 0.07, 0.03–0.12) and HbA1c (Q4 vs. Q1: β: 1.46 mmol/mol, 0.15–2.77). In T2DM (N = 599), higher meal irregularity was associated with elevated HbA1c levels (Q4 vs. Q1: β: 3.28, 0.19–6.37).

**Conclusion:**

These findings highlight the potential role of chrono-nutrition in glucose regulation, warranting further research for confirmation.

**Supplementary Information:**

The online version contains supplementary material available at 10.1007/s00394-026-03964-2.

## Introduction

Type 2 diabetes mellitus (T2DM) is a prevalent metabolic disorder with a rising incidence [1]. In 2021, the global prevalence of T2DM was estimated at 537 million adults, with projections indicating an increase to 783 million by 2045 [1]. When left unmanaged, T2DM can result in serious complications, including cardiovascular disease [2, 3]. T2DM typically develops gradually and is strongly associated with lifestyle factors such as physical inactivity and poor dietary habits [4]. Conversely, lifestyle interventions have been developed to delay or prevent T2DM by promoting a healthy diet and encouraging increased physical activity [3, 5].

Besides the quantity and quality of dietary intake, chrono-nutrition (the timing, frequency, and regularity of dietary intake) has also been hypothesized to play a role in the etiology of T2DM through its interplay with circadian rhythms [5, 6]. In more detail, circadian rhythms are endogenously generated approximately 24-h cycles in behavioral, physiological, and metabolic processes [7]. These rhythms are driven by internal molecular clocks, which regulate a substantial proportion of the genome and can be entrained by environmental cues [7]. Circadian clocks are present within most human organ and tissue cells. The circadian system is coordinated by the suprachiasmatic nucleus (SCN) in the hypothalamus, which functions as the central, light-entrained clock [5, 8, 9]. The SCN receives direct photic input via the retinohypothalamic tract and, in turn, coordinates rhythmicity in other brain regions and peripheral organs [5]. Importantly, peripheral clocks also possess self-sustaining oscillatory capacity and can entrain independently to non-photic cues or Zeitgebers, particularly dietary intake [10, 11]. When dietary intake occurs at regular and anticipated times, peripheral clocks remain aligned with the central clock, supporting optimal nutrient homeostasis [12]. In contrast, irregular or mistimed eating can desynchronize peripheral clocks from the central clock, leading to circadian misalignment [12]. In such cases, eating cues can shift the phase of peripheral clocks, altering their anticipated timing of metabolic processes [12]. Such disruptions to circadian rhythms can impair glycemic control by affecting beta cell function and insulin sensitivity, increasing the risk of developing T2DM [13, 14].

Previous research has shown that skipping breakfast is associated with lower insulin sensitivity and a higher risk of developing T2DM [15, 16]. A cross-sectional study demonstrated that late-night dinner consumption, which may lead to breakfast skipping, was associated with hyperglycemia [17]. Similarly, another study identified independent associations between these behaviors, and late-night dinner with poor glycemic control in individuals with T2DM [18]. Regarding meal frequency, a study found that men who ate three meals per day had a higher risk of developing T2DM compared to those who ate only once or twice daily [16]. Additionally, snacking, i.e. food consumption beyond the main meals, was associated with an increased T2DM risk [16]. In terms of meal irregularity, several studies suggest that irregular eating patterns negatively affect metabolic health, resulting in impaired glucose metabolism and an elevated risk of T2DM [19–21]. Lastly, both intervention and cross-sectional and prospective observational studies indicate that time-restricted eating or extending the overnight fasting period can benefit glucose metabolism and lower T2DM risk [5, 6, 22–24], consistent with findings that later and longer eating patterns are associated with higher cardiometabolic risk factors, including elevated fasting glucose [25].

Most of these studies investigated relatively small, homogenous samples, and only a few larger cohort studies have investigated chrono-nutrition in relation to glucose metabolism [24, 25]. Consequently, research in large populations including both healthy individuals and those with prediabetes or T2DM remains scarce. Therefore, in this study we aimed to investigate the associations between chrono-nutrition, specifically meal frequency, meal irregularity, and the length of the eating window, and glucose metabolism outcomes in healthy persons and those living with prediabetes or T2DM.

## Methods

### Study design and population

In this study, we used data from The Maastricht Study, an observational prospective population-based cohort study. The rationale and methodology have been described previously [26]. In brief, the study focuses on the etiology, pathophysiology, complications, and comorbidities of T2DM and is characterized by an extensive phenotyping approach. Eligible for participation were all individuals aged between 40 and 75 years and living in the southern part of the Netherlands. Participants were recruited through mass media campaigns and from the municipal registries and the regional Diabetes Patient Registry via mailings. Recruitment was stratified according to known T2DM status, with an oversampling of individuals with T2DM, for reasons of efficiency. The present report includes cross-sectional data of 3,467 participants who completed the baseline measurement between September 2017 at the inception of data collection on chrono-nutrition and December 2021, or were included based on their follow-up measurement between January 2022 and March 2024. The study has been approved by the institutional medical ethical committee (NL31329.068.10) and the Minister of Health, Welfare and Sports of the Netherlands (Permit 131088-105234-PG). All participants gave written informed consent.

### Data collection

#### Chrono-nutrition

Self-reported habitual information on participants’ meal frequency, timing, irregularity, and length of the eating window was collected using a single extensive custom-made chrono-nutrition questionnaire. This questionnaire captures habitual eating behavior during the past year, and all variables were therefore based on participants’ usual patterns rather than prospective logs or recalls. Participants also provided information on their habitual waking and sleeping times within this questionnaire. All questions were addressed separately for weekdays (Monday through Friday) and weekend days (Saturday and Sunday).

##### Meal frequency

The daily meal frequency for weekdays and weekend days was determined by adding the number of times participants consumed breakfast, lunch, dinner, and in-between snacks, including food and drinks, but excluding instances of consuming only water. A meal or eating occasion was defined as any intake of food or beverage (excluding water), regardless of calorie content, as even small amounts of bioactive compounds may act as cues for peripheral clocks [10]. This was determined separately for weekdays and weekends. This total was then divided by 5 for weekdays and by 2 for weekend days. To calculate the weighted average daily meal frequency of the entire week, the weekday frequency was multiplied by 5, the weekend frequency by 2, and the combined total was then divided by 7.

##### Meal irregularity

Participants indicated whether they consumed breakfast, lunch, dinner, and in-between snacks (morning, afternoon, and evening) regularly at the same clock-times on at least four out of five weekdays or at the same clock-time on weekend days (yes/no). They also reported how many weekdays and weekend days these meals and in-between snacks were consumed. Based on this information, a meal irregularity score variable was calculated. A value of 1 was assigned for each meal (breakfast, lunch, dinner) or snack period (morning, afternoon, and evening) that was consumed irregularly (i.e., not at the same clock-times on at least four weekdays or not at the same clock-time on weekend days, with timing deviating by at least 30 min). Similarly, a value of 1 was given if any of the meals or snack periods were skipped more than once during weekdays or once on the weekend. If meals or snack periods were always skipped, a value of 0 was assigned. Meal irregularity scores were calculated separately for weekdays and weekends, with scores ranging from 0 to 6 based on the six eating slots. To determine the overall weekly weighted meal irregularity, the weekday score was multiplied by 5, the weekend score by 2, and the combined total score was then divided by 7. Higher scores reflect greater irregularity. Additionally, breakfast skipping was defined based on self-reported frequency of breakfast consumption. Participants indicated on how many weekdays and weekend days they usually ate breakfast, and a dichotomous variable was created classifying individuals as breakfast skippers if they reported skipping breakfast on at least one of these days.

##### Meal timing and length of the eating window

To evaluate participants’ meal timing, several variables were established. First, the length of the eating window was calculated by determining the time between the first and last meal or snack of the day (excluding water) and expressed in decimal hours. Furthermore, the wake-to-first meal interval and last meal-to-bed interval were calculated as the number of hours between wake-up time and the first meal or snack (start of the eating window), and between the last meal or snack (end of the eating window) and bedtime, respectively. The eating windows as well as wake-to-first meal and last meal-to-bed intervals were assessed separately for weekdays and weekend days. To compute the overall weighted average values, the weekday values were multiplied by 5, the weekend values by 2, and the combined totals were then divided by 7.

#### Oral glucose tolerance test

To assess glucose metabolism status, all participants (except those using insulin) underwent a standardized seven-point oral glucose tolerance test (OGTT) following an overnight fast. Diabetes status was determined based on fasting plasma glucose and 2 h post-load glucose levels according to the World Health Organization’s 2006 criteria in combination with participants’ glucose-lowering medication use. Participants were classified into three categories: normal glucose metabolism (NGM) (fasting plasma glucose < 6.1 mmol/L and 2 h post-load glucose < 7.8 mmol/L), prediabetes (including impaired fasting glucose (IFG) (fasting plasma glucose ≥ 6.1—< 7.0 mmol/L and 2 h post-load glucose < 7.8 mmol/L) and impaired glucose tolerance (IGT) (fasting plasma glucose < 7.0 mmol/L and 2 h post-load glucose ≥ 7.8—< 11.1 mmol./L)), and T2DM (fasting plasma glucose ≥ 7.0 mmol/L or 2 h post-load glucose ≥ 11.1 mmol/L) [27]. Fasting plasma glucose (mmol/L) and hemoglobin A1c (HbA1c, mmol/mol) were measured in venous plasma samples collected after an overnight fast, while 2 h post-load glucose (mmol/L) was determined in venous plasma collected at 120 min post the 75-g glucose drink. Plasma glucose was measured with a standard enzymatic hexokinase reference method by an automatic analyzer (Beckman Synchron LX20, Beckman Coulter Inc., Brea, USA), whereas HbA1c was measured with ion-exchange high performance liquid chromatography (HPLC) (Variant tm II, Bio-Rad, Hercules, California, USA) [26].

#### Dietary intake

Habitual dietary intake of participants was assessed using the validated Maastricht FFQ (Food Frequency Questionnaire) [28]. Energy intake and specific nutrient values were calculated using the Dutch NEVO food composition table. Additionally, participants’ diet quality was evaluated by calculating an adherence score based on the Dutch Health Diet (DHD) recommendations for specific food groups [29]. The alcohol and coffee components were excluded, as alcohol was included as a separate variable, and data on filtered coffee consumption were unavailable. The total score ranged from 0 to 130, with higher scores indicating better diet quality.

#### Other relevant parameters

Weight and height were measured and recorded to the nearest 0.5 kg and 0.1 cm, respectively, to calculate BMI (kg/m^2^). Information on age and sex was obtained from study records, while data on education level, smoking status, physical activity (assessed using the CHAMPS questionnaire), employment status, marital status, and household income were gathered through self-reported questionnaires [26]. Information on irregular working hours, including shift work, was obtained from the chrono-nutrition questionnaire. Use of insulin, lipid-modifying, antihypertensive, and glucose-lowering medication was documented during an interview, including the name, dose, and frequency of medication use. Detailed descriptions of all measurement procedures have been published previously [26].

### Statistical analysis

Participant characteristics were summarized by glucose metabolism status, using either mean (SD) or median (IQR) for continuous variables as appropriate, and number (percentage) for categorical variables.

Multinomial logistic regression models were used to estimate odds ratios (ORs) and 95% confidence intervals (CIs) for associations between chrono-nutrition variables and glucose metabolism status (prediabetes or T2DM vs. NGM). Multiple linear regression models, stratified by glucose metabolism status because of significant interaction terms, were used to assess associations of chrono-nutrition with continuous measures of glucose metabolism (fasting plasma glucose, 2 h post-load glucose, HbA1c). Associations were adjusted for age, sex, education level (low, middle, high), BMI (kg/m^2^), total physical activity (hours/week), smoking status (never, former, current), energy intake (kcal/day), alcohol intake (gram/day), and dietary quality (DHD-index). Stratified linear models within participants with T2DM were additionally adjusted for glucose-lowering medication use (yes/no). Chrono-nutrition variables were analyzed continuously and categorically (using quartiles based on overall population distributions). In all analyses, model assumptions were checked and found to be not violated.

Interaction terms for sex and employment status were tested in all models. Stratified analyses were conducted in case of significant interactions (*P* < 0.05). Sensitivity analyses investigated chrono-nutrition separately for weekdays and weekends, included additional covariates (shift work, marital status, household income, and employment status) to assess consistency and potential residual confounding, and investigated newly diagnosed T2DM (based on the OGTT results without prior diagnosis or diabetes medication use) as a separate outcome to account for potential dietary changes post-diagnosis. In addition, to align with the updated WHO diagnostic criteria, analyses were repeated after reclassifying individuals with HbA1c ≥ 6.5% (48 mmol/mol) as having T2DM. Furthermore, analyses were repeated adjusting for pack-years of smoking instead of smoking status in the subsample of participants with available data on both. Additionally, all analyses were repeated without adjustment for BMI, given that BMI may lie on the causal pathway and could therefore act as a mediator. All statistical analyses were conducted using Stata 16.0 (StataCorp LLC) and IBM SPSS Statistics 27 (IBM, Armonk, NY, USA) with statistical significance set at *P* < 0.05 (two-sided).

## Results

### Characteristics of the study population

Of the 3,467 participants included in this study, 2,264 had NGM, 604 had prediabetes, and 599 had T2DM. Participants were on average (mean (SD)) 64 (9) years with 50.9% women, and the mean BMI of participants was 26.6 (4.3) kg/m^2^. Additionally, 40.4% of participants were employed, of whom 13.6% at least sometimes worked irregular hours or shifts (Table 1).

**Table 1:** Sociodemographic, lifestyle, and clinical characteristics of The Maastricht Study participants included in the current study according to glucose metabolism status

Characteristics	Total population (N = 3467)^1^	Normal glucose metabolism (N = 2264)^1^	Prediabetes (N = 604)^1^	Type 2 diabetes mellitus (N = 599)^1^
Age, yr	64 ± 9	62 ± 9	65 ± 8	67 ± 8
Women, %	50.9	57.0	46.0	32.9
BMI, kg/m^2^	26.6 ± 4.3	25.6 ± 3.8	28.0 ± 4.3	29.1 ± 4.4
BMI categorical, %				
Normal: < 25.0 kg/m^2^	38.9	49.1	23.0	16.7
Overweight: 25.0 – 29.9 kg/m^2^	42.1	39.5	48.3	45.2
Obese: ≥ 30 kg/m^2^	19.0	11.4	28.6	38.1
Education, %				
Low	24.1	20.8	29.3	31.2
Medium	28.4	28.4	27.6	29.4
High	47.5	50.8	43.0	39.4
Smoking, %				
Never	42.2	46.2	36.1	33.2
Former	50.2	46.4	56.3	58.4
Current	7.6	7.5	7.6	8.3
Total physical activity, h/week	12.8 (8.3 – 18.5)	13.3 (9.0 – 19.0)	12.3 (8.0 – 18.4)	11.0 (6.5 – 16.0)
Dietary intake				
Energy, kcal/day	1922 ± 585	1945 ± 585	1916 ± 576	1839 ± 586
Alcohol intake, g/day	6.8 (1.4 – 14.2)	6.7 (1.6 – 13.4)	8.8 (1.7 – 17.8)	5.3 (0.7 – 15.1)
DHD-index (excl. alcohol)	79.3 ± 15.0	80.9 ± 14.6	77.4 ± 15.3	75.1 ± 15.3
HbA1c, mmol/mol	36 (34 – 40)	35.1 ± 3.5	37.7 ± 4.0	50.7 ± 10.4
Fasting plasma glucose, mmol/L	5.4 (5.0 – 6.0)	5.2 ± 0.4	5.8 ± 0.6	8.0 ± 2.0
2 h post-load glucose, mmol/L	6.0 (4.9 – 7.9)	5.4 ± 1.2	8.1 ± 1.6	14.6 ± 4.0
Glucose lowering medication, % yes	10.4	0.0	0.0	60.3
Hypertension medication, % yes	35.4	24.0	44.2	69.4
Lipid-modifying medication, % yes	25.7	14.7	29.3	63.6
Household income, euros net per month (considering household size)	2449 ± 916	2493 ± 913	2425 ± 904	2302 ± 928
Employment status, % employed	40.4	46.7	32.1	25.4
Marital status, % married	72.7	71.7	74.5	74.6
Shift work (working irregular hours, % yes (among employed)	13.6	14.2	14.4	8.6

*BMI* Body mass index; *DHD* Dutch Healthy Diet; *HbA1c* Glycohemoglobin A1c. Values are presented as percentage, mean ± standard deviation, or median (IQR, 25th – 75th percentile) according to their distribution

^1^Percentages may not add up to 100 due to rounding

### Descriptives of chrono-nutrition variables

The mean daily meal frequency was 7.3 (4.9) among participants with NGM, 6.9 (4.4) in those with prediabetes, and 6.9 (4.0) in T2DM (Table 2). The average length of the eating window was 12.4 (1.9) hours for the total population. Weekdays and weekends showed small differences in average eating window, largely attributable to earlier breakfast timing. The last meal-to-bed interval was 2.6 (1.5) hours for the total population. Meal irregularity values were 2.4 (1.5) in NGM and 2.2 (1.5) among those with prediabetes and T2DM. Finally, 13.0% of participants reported skipping breakfast at least once per week, with similar proportions across glucose metabolism groups (Table 2). Differences in the number of participants with meal frequency, eating windows, meal irregularity, and breakfast skipping reflect exclusions due to incomplete data.

**Table 2:** Descriptive analyses of chrono-nutrition variables in the study population of The Maastricht Study participants according to glucose metabolism status

Characteristics	Total population (N = 3467)	Normal glucose metabolism (N = 2264)	Prediabetes (N = 604)	Type 2 diabetes mellitus (N = 599)
Meal frequency, average total meals per day (n = 2224)				
Total	7.2 ± 4.7	7.3 ± 4.9	6.9 ± 4.4	6.9 ± 4.0
Weekdays	7.4 ± 5.6	7.5 ± 5.8	7.1 ± 5.3	7.0 ± 4.5
Weekend days	6.7 ± 3.7	6.8 ± 3.7	6.3 ± 3.7	6.5 ± 3.8
Length of the eating window, hours (n = 2771)				
Total	12.4 ± 1.9	12.5 ± 1.8	12.5 ± 2.1	12.2 ± 2.0
Weekdays	12.6 ± 2.1	12.6 ± 2.0	12.6 ± 2.3	12.3 ± 2.1
Weekend days	12.1 ± 2.1	12.2 ± 2.0	12.1 ± 2.1	11.8 ± 2.3
Wake-to-first meal interval, hours (n = 2665)				
Total	0.6 ± 0.8	0.6 ± 0.8	0.6 ± 0.8	0.5 ± 0.7
Weekdays	0.6 ± 0.9	0.6 ± 0.9	0.6 ± 0.9	0.5 ± 0.7
Weekend days	0.6 ± 0.8	0.6 ± 0.8	0.5 ± 0.7	0.6 ± 0.8
Last meal-to-bed interval, hours (n = 2724)				
Total	2.6 ± 1.5	2.5 ± 1.5	2.6 ± 1.6	2.8 ± 1.7
Weekdays	2.6 ± 1.7	2.5 ± 1.6	2.6 ± 1.7	2.8 ± 1.8
Weekend days	2.6 ± 1.7	2.5 ± 1.6	2.6 ± 1.6	2.8 ± 1.9
Meal irregularity, higher score indicating more irregularity (range 0 – 6) (n = 2016)				
Total	2.4 ± 1.5	2.4 ± 1.5	2.2 ± 1.5	2.2 ± 1.5
Weekdays	2.2 ± 1.6	2.2 ± 1.6	2.0 ± 1.6	2.1 ± 1.6
Weekend days	2.9 ± 1.8	3.0 ± 1.8	2.7 ± 1.8	2.5 ± 1.7
Breakfast skipping at least once per week, % yes (n = 2952)				
	13.0	13.3	14.0	10.8

### Adjusted associations of meal frequency with glucose metabolism

In the multinomial logistic regression models, no significant associations were found between daily meal frequency and the presence of prediabetes or T2DM compared to NGM, although highest meal frequencies tended to be associated with higher odds of having T2DM (Q4 vs Q1; OR: 1.40, 0.96–2.06) (Table 3). Similarly, no significant associations were observed between daily meal frequency and fasting plasma glucose, 2 h post-load glucose, or HbA1c levels (Table 3).

**Table 3:** Confounder-adjusted associations between meal frequency and glucose metabolism outcomes within participants of The Maastricht Study

Meal frequency	Q1^1,2^	Q2	Q3	Q4	Continuous, Per meal increase
	OR (95% CI)	OR (95% CI)	OR (95% CI)	OR (95% CI)	OR^3^ (95% CI)
Multinominal logistic regression – Odds of having prediabetes/T2DM compared to NGM					
Prediabetes vs NGM	REF	0.96 (0.68, 1.34)	0.70 (0.49, 1.01)	1.00 (0.70, 1.42)	1.00 (0.97, 1.02)
T2DM vs NGM	REF	1.05 (0.71, 1.54)	0.88 (0.59, 1.32)	1.40 (0.94, 2.06)	1.01 (0.98, 1.04)
Linear regression – Stratified by glucose metabolism status^5^					
	β (95% CI)	β (95% CI)	β (95% CI)	β (95% CI)	β^4^ (95% CI)
Fasting plasma glucose concentration (mmol/L)					
NGM	REF	0.03 (−0.03, 0.08)	0.00 (−0.05, 0.06)	−0.02 (−0.08, 0.04)	0.00 (−0.01, 0.00)
Prediabetes	REF	0.07 (−0.09, 0.23)	−0.05 (−0.23, 0.13)	0.15 (−0.02, 0.32)	0.01 (−0.01, 0.02)
T2DM	REF	−0.06 (−0.63, 0.51)	−0.20 (−0.81, 0.41)	0.21 (−0.36, 0.77)	0.04 (−0.01, 0.09)
2 h post-load plasma glucose concentration (mmol/L)					
NGM	REF	−0.01 (−0.18, 0.15)	−0.08 (−0.24, 0.08)	−0.11 (−0.28, 0.06)	−0.01 (−0.02, 0.01)
Prediabetes	REF	0.06 (−0.43, 0.54)	0.41 (−0.13, 0.94)	−0.23 (−0.74, 0.29)	−0.02 (−0.06, 0.02)
T2DM	REF	0.67 (−0.66, 2.01)	−0.94 (−2.28, 0.40)	1.03 (−0.22, 2.28)	0.03 (−0.09, 0.15)
HbA1c concentration (mmol/mol)					
NGM	REF	−0.12 (−0.59, 0.35)	0.15 (−0.32, 0.61)	0.33 (−0.16, 0.82)	0.03 (0.00, 0.06)
Prediabetes	REF	0.06 (−1.06, 1.18)	0.71 (−0.52, 1.93)	0.09 (−1.09, 1.28)	−0.05 (−0.14, 0.05)
T2DM	REF	1.91 (−0.79, 4.61)	−1.81 (−4.70, 1.08)	1.45 (−1.24, 4.14)	0.17 (−0.09, 0.42)

*Q* Quartile; OR, odds ratio; CI, confidence interval; REF, reference; NGM, normal glucose metabolism; *T2DM* Type 2 diabetes mellitus; *HbA1c* Glycohemoglobin A1c

^1^Q1 ranged from ≤ 4.00 average meals daily, Q2 ranged from 4.07 – 6.14 meals daily, Q3 ranged from 6.21 – 8.86 meals daily, and Q4 ranged from ≥ 8.93 meals daily

^2^All associations were adjusted for age, sex, education level, BMI, physical activity levels, smoking status, energy intake, alcohol intake and dietary quality. Linear regression models stratified within participants with T2DM were additionally adjusted for the use of glucose lowering medication

^3^The OR indicates the odds for having prediabetes/T2DM compared to the reference category of NGM if meal frequency increases with one

^4^The β indicates the average increase/decrease in the outcome variable if meal frequency increases with one

^5^The number of participants included with a normal glucose metabolism was 1582, whereas 346 participants had prediabetes, and 296 T2DM

### Adjusted associations of the length of the eating window with glucose metabolism

Participants with a longer eating window had higher odds of prediabetes compared to NGM in multinomial logistic regression models (OR per hour: 1.07; 1.01, 1.13) (Table 4). Specifically, those with an eating window longer than 12.7 h (Q3 and Q4) had increased odds compared to those with an eating window shorter than 11.2 h (Q1). No significant associations were found for T2DM compared to NGM. In participants with NGM (N = 1849), an eating window longer than 13.8 h (Q4) was significantly associated with higher 2 h post-load plasma glucose concentrations compared to Q1 (< 11.18 h) (β: 0.16 mmol/L; 0.00, 0.31). No significant associations were observed for other glucose metabolism groups or for fasting plasma and HbA1c levels (Table 4).

**Table 4:** Confounder-adjusted associations between the length of the eating window and glucose metabolism outcomes within participants of The Maastricht Study

Length of eating window	Q1^1,2^	Q2	Q3	Q4	Continuous, Per hour increase
	OR (95% CI)	OR (95% CI)	OR (95% CI)	OR (95% CI)	OR^3^ (95% CI)
Multinominal logistic regression – Odds of having prediabetes/T2DM compared to NGM					
Prediabetes vs NGM	REF	0.82 (0.60, 1.12)	1.20 (0.89, 1.63)	1.25 (0.92, 1.70)	**1.07 (1.01, 1.13)**
T2DM vs NGM	REF	0.88 (0.64, 1.21)	1.04 (0.75, 1.45)	1.18 (0.85, 1.65)	1.03 (0.97, 1.10)
Linear regression – Stratified by glucose metabolism status^5^					
	β (95% CI)	β (95% CI)	β (95% CI)	β (95% CI)	β^4^ (95% CI)
Fasting plasma glucose concentration (mmol/L)					
NGM	REF	−0.01 (−0.06, 0.04)	0.00 (−0.05, 0.06)	0.01 (−0.04, 0.07)	0.00 (−0.01, 0.01)
Prediabetes	REF	−0.05 (−0.20, 0.10)	0.03 (−0.11, 0.17)	0.08 (−0.07, 0.22)	0.02 (−0.01, 0.04)
T2DM	REF	−0.32 (−0.80, 0.16)	−0.31 (−0.80, 0.18)	−0.42 (−0.92, 0.08)	−0.06 (−0.15, 0.03)
2 h post-load plasma glucose concentration (mmol/L)					
NGM	REF	0.13 (−0.01, 0.28)	0.09 (−0.06, 0.24)	**0.16 (0.00, 0.31)**	0.03 (0.00, 0.06)
Prediabetes	REF	0.24 (−0.19, 0.67)	0.30 (−0.12, 0.72)	0.13 (−0.30, 0.57)	0.04 (−0.04, 0.11)
T2DM	REF	−0.06 (−1.11, 0.98)	0.04 (−1.01, 1.09)	−0.37 (−1.45, 0.70)	0.03 (−0.16, 0.23)
HbA1c concentration (mmol/mol)					
NGM	REF	0.31 (−0.12, 0.75)	0.20 (−0.24, 0.64)	0.13 (−0.32, 0.58)	0.01 (−0.08, 0.09)
Prediabetes	REF	−0.57 (−1.24, 0.78)	−0.23 (−1.24, 0.78)	−0.35 (−1.40, 0.69)	−0.06 (−0.24, 0.13)
T2DM	REF	1.58 (−0.82, 3.97)	1.60 (−0.87, 4.07)	−0.35 (−2.84, 2.15)	0.13 (−0.34, 0.59)

Bold values indicate statistically significant associations (*P* < 0.05)

*Q* Quartile; *OR* odds ratio; *CI* Confidence interval; *REF* Reference; *NGM* Normal glucose metabolism; *T2DM* Type 2 diabetes mellitus; *HbA1c* Glycohemoglobin A1c

^1^Q1 ranged from ≤ 11.18 h, Q2 ranged from 11.21 – 12.68 h, Q3 ranged from 12.71 – 13.75 h, and Q4 ranged from ≥ 13.79 h

^2^All associations were adjusted for age, sex, education level, BMI, physical activity levels, smoking status, energy intake, alcohol intake and dietary quality. Linear regression models stratified within participants with T2DM were additionally adjusted for the use of glucose lowering medication

^3^The OR indicates the odds for having prediabetes/T2DM compared to the reference category of NGM if the eating window increases with one hour

^4^The β indicates the average increase/decrease in the outcome variable if the eating window increases with one hour

^5^The number of participants included with a normal glucose metabolism was 1849, whereas 479 participants had prediabetes, and 443 T2DM

Among participants with NGM, a longer wake-to-first meal interval was associated with higher HbA1c levels in Q2 vs Q1 (β: 0.55 mmol/mol; 0.10, 1.00) and Q4 vs Q1 (β: 0.55 mmol/mol; 0.11, 0.99). In participants with prediabetes, a similar pattern was observed for Q2 vs Q1 (β: 1.15 mmol/mol; 0.08. 2.22). For the last meal-to-bed interval, participants with T2DM showed lower 2 h post-load plasma glucose levels in Q2 vs Q1 (β: −1.17 mmol/L; −2.26, −0.07). No other associations were found for either of these intervals in relation to fasting plasma glucose, 2 h post-load glucose, HbA1c, or the odds of having prediabetes or T2DM compared to NGM (Supplementary Table 1).

### Adjusted associations of meal irregularity with glucose metabolism

No significant associations were observed in the multinomial logistic regression models between meal irregularity and the presence of prediabetes or T2DM, compared to NGM (Table 5). Notably, among participants with prediabetes (N = 306), higher meal irregularity scores were associated with higher fasting plasma glucose (β: 0.07 mmol/L, 0.03–0.12) and HbA1c (Q4 vs Q1; β: 1.46, 0.15–2.77) In participants with T2DM (N = 253), similar but numerically stronger associations for HbA1c were observed (Q3 vs Q1; β: 3.55 mmol/mol; 0.66, 6.44, Q4 vs Q1; β: 3.28; 0.19, 6.37). In participants with NGM, a meal irregularity score between 2.4 and 3.4 (Q3) was significantly associated with lower 2 h post-load glucose levels compared to scores lower than 1.2 (Q1) (β: −0.18 mmol/L; −0.35, −0.01) (Table 5).

**Table 5:** Confounder-adjusted associations between meal irregularity and glucose metabolism outcomes within participants of The Maastricht Study

Meal irregularity score^1^	Q1^2,3^	Q2	Q3	Q4	Continuous, Per one increase
	OR (95% CI)	OR (95% CI)	OR (95% CI)	OR (95% CI)	OR^4^ (95% CI)
Multinominal logistic regression – Odds of having prediabetes/T2DM compared to NGM					
Prediabetes vs NGM	REF	0.96 (0.68, 1.37)	0.93 (0.64, 1.35)	0.83 (0.57, 1.21)	0.95 (0.86, 1.03)
T2DM vs NGM	REF	0.91 (0.61, 1.37)	1.15 (0.76, 1.73)	0.92 (0.59, 1.42)	1.01 (0.91, 1.12)
Linear regression – Stratified by glucose metabolism status^6^					
	β (95% CI)	β (95% CI)	β (95% CI)	β (95% CI)	β^5^ (95% CI)
Fasting plasma glucose concentration (mmol/L)					
NGM	REF	0.02 (−0.03, 0.08)	0.00 (−0.06, 0.06)	0.02 (−0.04, 0.08)	0.00 (−0.01, 0.02)
Prediabetes	REF	0.12 (−0.05, 0.29)	**0.25 (0.08, 0.43)**	**0.24 (0.05, 0.42)**	**0.07 (0.03, 0.12)**
T2DM	REF	0.12 (−0.50, 0.74)	0.08 (−0.53, 0.70)	−0.04 (−0.69, 0.62)	−0.02 (−0.17, 0.13)
2 h post-load plasma glucose concentration (mmol/L)					
NGM	REF	−0.03 (−0.19, 0.13)	**−0.18 (**−**0.35,** −**0.01)**	−0.16 (−0.33, 0.01)	−**0.05 (**−**0.09,** −**0.01)**
Prediabetes	REF	−0.07 (−0.57, 0.44)	−0.30 (−0.83, 0.24)	−0.45 (−1.01, 0.10)	−0.12 (−0.25, 0.02)
T2DM	REF	−0.70 (−2.06, 0.66)	0.04 (−1.36, 1.45)	−0.61 (−2.08, 0.85)	−0.19 (−0.53, 0.15)
HbA1c concentration (mmol/mol)					
NGM	REF	0.03 (−0.43, 0.49)	0.00 (−0.48, 0.48)	0.31 (−0.16, 0.78)	0.10 (−0.02, 0.21)
Prediabetes	REF	−0.23 (−1.43, 0.96)	0.06 (−1.21, 1.32)	**1.46 (0.15, 2.77)**	**0.37 (0.06, 0.69)**
T2DM	REF	−0.01 (−2.92, 2.90)	**3.55 (0.66, 6.44)**	**3.28 (0.19, 6.37)**	**0.79 (0.07, 1.51)**

Bold values indicate statistically significant associations (*P* < 0.05)

Q, quartile; OR, odds ratio; CI, confidence interval; REF, reference; NGM, normal glucose metabolism; T2DM, type 2 diabetes mellitus; HbA1c, glycohemoglobin A1c

^1^Higher scores for meal irregularity indicate a higher meal irregularity, with a score ranging from 0 (highly regular) to 6 (highly irregular)

^2^Q1 ranged from 0.00 – 1.21 average meal irregularity, Q2 ranged from 1.29 – 2.29 meal irregularity, Q3 ranged from 2.36 – 3.36 meal irregularity, and Q4 ranged from 3.43 – 6.00 meal irregularity

^3^All associations were adjusted for age, sex, education level, BMI, physical activity levels, smoking status, energy intake, alcohol intake and dietary quality. Linear regression models stratified within participants with T2DM were additionally adjusted for the use of glucose lowering medication

^4^The OR indicates the odds for having prediabetes/T2DM compared to the reference category of NGM if the meal irregularity score increases with one

^5^The β indicates the average increase/decrease in the outcome variable if the meal irregularity score increases with one

^6^The number of participants included with a normal glucose metabolism was 1457, whereas 306 participants had prediabetes, and 253 T2DM

### Adjusted associations of breakfast skipping with glucose metabolism

Skipping breakfast at least once per week was not significantly associated with prediabetes or T2DM compared to NGM in multinomial logistic regression models (Table 6). However, it was associated with higher fasting plasma glucose levels in participants with NGM (N = 2013), as shown in the linear regression model (β: 0.09 mmol/L; 0.04, 0.14). An inverse association between breakfast skipping and 2 h post-load plasma glucose was found among participants with prediabetes (β: -0.50 mmol/L; -0.96, -0.05).

**Table 6:** Confounder-adjusted associations between breakfast skipping and glucose metabolism outcomes within participants of The Maastricht Study

	Breakfast skipping, yes
	OR^1,2^ (95% CI)
Multinominal logistic regression – Odds of having prediabetes/T2DM compared to NGM	
Prediabetes vs NGM	1.12 (0.82, 1.53)
T2DM vs NGM	0.84 (0.59, 1.20)
Linear regression – Stratified by glucose metabolism status^3^	
	β^4^ (95% CI)
Fasting plasma glucose concentration (mmol/L)	
NGM	**0.09 (0.04, 0.14)**
Prediabetes	0.05 (−0.10, 0.21)
T2DM	0.13 (−0.40, 0.65)
2 h post-load plasma glucose concentration (mmol/L)	
NGM	−0.10 (−0.25, 0.05)
Prediabetes	**−0.50 (−0.96, −0.05)**
T2DM	−0.54 (−1.75, 0.68)
HbA1c concentration (mmol/mol)	
NGM	0.01 (−0.42, 0.43)
Prediabetes	0.85 (−0.19, 1.90)
T2DM	1.31 (−1.18, 3.81)

Bold values indicate statistically significant associations (*P* < 0.05)

*Q* Quartile; *OR* Odds ratio; *CI* Confidence interval; *REF* Reference; *NGM* Normal glucose metabolism; T2DM, type 2 diabetes mellitus; HbA1c, glycohemoglobin A1c

^1^All associations were adjusted for age, sex, education level, BMI, physical activity levels, smoking status, energy intake, alcohol intake and dietary quality. Linear regression models stratified within participants with T2DM were additionally adjusted for the use of glucose lowering medication

^2^The OR indicates the odds for having prediabetes/T2DM compared to the reference category of NGM if the breakfast is skipped at least once per week

^3^The number of participants included with a normal glucose metabolism was 2013, whereas 477 participants had prediabetes, and 462 T2DM

^4^The β indicates the average increase/decrease in the outcome variable if the breakfast is skipped at least once per week

### Associations in the total study population

When analyses were conducted in the total study population without stratification by glucose metabolism status, the pattern of associations mostly resembled those observed in participants with NGM, who constituted the largest proportion of the study population. Effect estimates were generally attenuated compared to those observed in individuals with prediabetes or T2DM. Notably, a higher meal irregularity score remained significantly associated with higher HbA1c concentrations and lower 2 h post-load glucose, and breakfast skipping remained associated with higher fasting plasma glucose levels in the total study population.

### Secondary analyses

No significant effect modification by sex or employment status was observed for the associations between chrono-nutrition variables and glucose metabolism outcomes. Furthermore, there were no major differences in associations with separate weekday and weekend chrono-nutrition values compared to the main results (Supplementary Fig. 1). Additionally adjusting for shift work, marital status, household income, or employment status yielded similar findings. When adjusting for pack-years of smoking instead of smoking status, results were in the same direction and the conclusions remained unchanged. Though, associations between meal frequency and fasting plasma glucose were slightly stronger in individuals with prediabetes and T2DM, whereas associations with HbA1c in prediabetes were slightly attenuated. Reclassifying individuals with HbA1c ≥ 6.5% as having T2DM in accordance with the updated WHO criteria did not materially alter the results, and the main conclusions remained unchanged. Repeating the analyses without adjustment for BMI did not change the direction or significance of the associations. However, associations with fasting plasma glucose and HbA1c were generally slightly stronger, whereas those with 2 h post-load glucose were slightly attenuated. Associations with only newly diagnosed T2DM (N = 238) compared to the total population with T2DM were generally similar in multinomial logistic regression models (Supplementary Fig. 2).

## Discussion

This is the first cross-sectional population-based study investigating the association of chrono-nutrition with glucose metabolism status across the range from normal glucose to prediabetes to T2DM. We found that longer eating windows, reflecting a shorter overnight fast, were associated with higher odds of prediabetes. Interestingly, we found that associations between chrono-nutrition and fasting plasma glucose, 2 h post-load glucose, and HbA1c concentrations differed somewhat by glucose metabolism status. When analyses were conducted in the total study population without stratification, associations were generally weaker and most closely resembled those observed in normoglycemic individuals, likely reflecting their larger representation in the study population. In participants with prediabetes, higher meal irregularity was associated with higher fasting plasma glucose and HbA1c, while breakfast skipping was associated with lower 2 h post-load glucose. In participants with T2DM, higher meal irregularity was associated with higher HbA1c levels. In participants with NGM, breakfast skipping was associated with higher fasting plasma glucose.

Our findings showed no significant associations between meal frequency and glucose metabolism outcomes. A previous randomized cross-over study showed that fewer meals per day (two vs six) resulted in greater decreases in fasting plasma glucose in patients with T2DM [30]. However, in line with our findings, reviews reported limited evidence for an impact of meal frequency on glucose control in short-term intervention studies, ranging from a couple of weeks up to one year in both healthy and glucose impaired adults [31, 32]. For meal irregularity, we observed higher fasting plasma glucose concentrations in participants with prediabetes who had greater irregularity, and higher HbA1c concentrations in both participants with prediabetes and those with T2DM. Previous observational studies have linked meal irregularity, including breakfast skipping, to impaired glucose metabolism [19], elevated HbA1c [33], and higher fasting plasma glucose concentrations in both healthy individuals and those with T2DM [34, 35]. Large prospective studies have suggested that breakfast skipping increases the risk of T2DM [36]. Similarly, we found a significant association between breakfast skipping and higher fasting plasma glucose, albeit primarily among participants with NGM. However, the association with 2 h post-load glucose was in the opposite direction. The strength of our findings may have been limited by our definition of breakfast skipping (≥ 1 day/week) and its low prevalence (13%) in our study population, as stronger associations may have been observed among participants who skipped breakfast more regularly.

We found that longer eating windows were associated with higher odds of prediabetes and T2DM compared to shorter eating windows, although this was only significant for prediabetes. These findings support previous literature suggesting that shorter eating windows, characterized by longer overnight fasting, improve glycemic control [6] and reduce T2DM risk [24]. Additionally, reviews have shown that shorter eating windows, as seen in intermittent fasting, are related to lower fasting plasma glucose and HbA1c levels [22, 23, 37]. While this may seem contradictory to our findings on breakfast skipping, which may result in a longer overnight fast and was associated with higher fasting plasma glucose among participants with a NGM, this discrepancy may be explained by differences in overall meal timing. Specifically, a longer eating window could indicate late-night eating, which has been associated with impaired glucose metabolism [17, 18]. This suggests that not only the length of the overnight fast, but in particular the timing of the last meal, may play a crucial role. In our study, we did not observe many significant associations between the length of the eating window and fasting plasma glucose or HbA1c levels. This discrepancy with findings of previous literature could stem from the range of eating windows in our population, where the average was approximately 12 h, differing from most intermittent fasting studies with more extreme lengths of eating windows, such as 8 h or less [22, 23].

Important to note is that some associations showed opposing trends between fasting plasma glucose and HbA1c on the one hand and 2 h post-load glucose on the other, albeit that these differences were mostly not statistically significant. This divergence likely reflects the distinct physiological mechanisms underlying these measures. More specifically, fasting plasma glucose is influenced by hepatic glucose production, HbA1c reflects long-term glycemic control over 2–3 months, and 2 h post-load glucose primarily captures glucose clearance and peripheral insulin sensitivity following an acute glucose challenge [38]. These opposing trends were predominantly observed for variables indicating irregularity of meals and were most apparent in the prediabetes group. When the prediabetes group was further divided into impaired fasting glucose (IFG) and impaired glucose tolerance (IGT) subgroups, almost all associations with 2 h post-load glucose became smaller and non-significant in both subgroups (Supplementary Table 2). This suggests that the seemingly discrepant associations observed for 2 h post-load glucose may potentially have been due to chance. This interpretation is further supported by previous research showing that breakfast skipping increases postprandial hyperglycemia after lunch and dinner in patients with T2DM [39]. For fasting plasma glucose and HbA1c, stronger associations were observed for individuals with IGT compared to those with IFG (Supplementary Table 2), indicating that glucose metabolism status may play a role in these relationships. This aligns with the overall findings of this study.

The magnitude of the regression coefficients observed in this study was modest. For the investigated chrono-nutrition measures (e.g., per additional meal or eating occasion, or per hour longer eating window), continuous betas ranged as follows: fasting plasma glucose: −0.06 to 0.13 mmol/L; 2 h post-load glucose: −0.54 to 0.04 mmol/L; HbA1c: −0.06 to 1.31 mmol/mol. These values are relatively small compared to the normal values of < 5.6 mmol/L for fasting plasma glucose, < 7.8 mmol/L for 2 h post-load glucose, and < 39 mmol/mol for HbA1c, as well as to the minimal clinically important difference [25, 40]. However, changing multiple aspects of chrono-nutrition or achieving larger improvements could yield more clinically meaningful effects. While it is well established that healthier dietary practices could improve glucose metabolism [3–5], considering the temporal aspects of eating behavior may provide an additional benefit. Future prospective and intervention studies targeting these temporal dimensions could potentially further enhance the control of blood glucose levels to prevent complications.

Our study has several strengths and limitations. A key strength is the comprehensive assessment of chrono-nutrition, since chrono-nutrition aspects are often not measured by standard instruments (e.g. FFQs). Combined with our large population-based sample, including participants with NGM, prediabetes, and an oversampled group of T2DM, this allowed us to provide novel insights into associations across all three groups. Face and content validity were evaluated among individuals from the target population and indicated that the chrono-nutrition questionnaire used was understandable and accurately reflected their eating habits. Formal validation against dietary records is still ongoing. Furthermore, detailed data on sociodemographic, clinical, and lifestyle factors enabled robust adjustment for potential confounders, limiting the potential of residual confounding. A limitation is that the cross-sectional design of our study does not allow for making causal inferences. In fact, participants with health issues (partly) related to impaired glucose metabolism may have already improved the quality of their eating habits based on their condition or medical advice, rather than their chrono-nutrition behaviors being the cause of their impaired glucose metabolism. Although this may have played a role for people with established, i.e. known T2DM, most participants were unaware of their glucose impairment, limiting the risk of reverse causality. This is further supported by the similar associations found in multinomial logistic regression models with only newly diagnosed T2DM participants, who were unaware of their T2DM status.

In conclusion, longer eating windows are cross-sectionally associated with higher odds of prediabetes compared to NGM. Associations between chrono-nutrition and glucose metabolism outcomes vary by glucose metabolism status. Higher meal irregularity is linked to elevated fasting plasma glucose in prediabetes and with higher HbA1c levels in prediabetes and T2DM, whereas breakfast skipping is associated with higher fasting plasma glucose in individuals with NGM. Future longitudinal research is needed to confirm these findings and further investigate underlying mechanisms.

## Supplementary Information

Below is the link to the electronic supplementary material.

Supplementary fig 1 Comparison plots illustrating the differences in adjusted continuous associations between weighted (week & weekend) chrono-nutrition variables and outcomes, as well as separate associations for chrono-nutrition variables based on week and weekend days alone. Abbreviations: CI, confidence interval; OR, odds ratio; NGM, normal glucose metabolism; adj., adjusted; T2DM, type 2 diabetes mellitus; FPG, fasting plasma glucose; PLG; 2-h post-load glucose; HbA1c, hemoglobin A1c. Odds ratios were estimated using multinomial logistic regression models, while associations with FPG, PLG, and HbA1c were analyzed using linear regression models. (PDF 895 kb)

Supplementary fig 2 Comparison plots illustrating the differences in adjusted continuous associations between chrono-nutrition and outcomes, for the whole T2DM group (as in main analyses) compared to only participants with newly diagnosed T2DM. Abbreviations: CI, confidence interval; OR, odds ratio; adj., adjusted; T2DM, type 2 diabetes mellitus; FPG, fasting plasma glucose; PLG; 2-h post-load glucose; HbA1c, hemoglobin A1c. Odds ratios were estimated using multinomial logistic regression models, while associations with FPG, PLG, and HbA1c were analyzed using linear regression models. (PDF 246 kb)

Supplementary file3 (DOCX 33 kb)

## Data Availability

The data of this study derives from The Maastricht Study, but restrictions apply to the availability of these data, which were used under license for the current study. Data are, however, available from the authors upon reasonable request and with permission of The Maastricht Study management team (um-researchdms@maastrichtuniversity.nl).

## Abbreviations

DHD: Dutch healthy diet
FFQ: Food frequency questionnaire
HbA1c: Hemoglobin A1c
IFG: Impaired fasting glucose
IGM: Impaired glucose metabolism
IGT: Impaired glucose tolerance
IQR: Interquartile range
NGM: Normal glucose metabolism
OGTT: Oral glucose tolerance test
OR: Odds ratio
SCN: Suprachiasmatic nucleus
SD: Standard deviation
T2DM: Type 2 diabetes mellitus
