# Supplementary figures and images for: Associations between chrono-nutrition and glucose metabolism across levels of glucose impairment: The Maastricht Study
Source: Eur J Nutr. 2026 Apr 21;65(4):119. doi: 10.1007/s00394-026-03964-2 (PMC13099856; doi:10.1007/s00394-026-03964-2)

Comparison plot

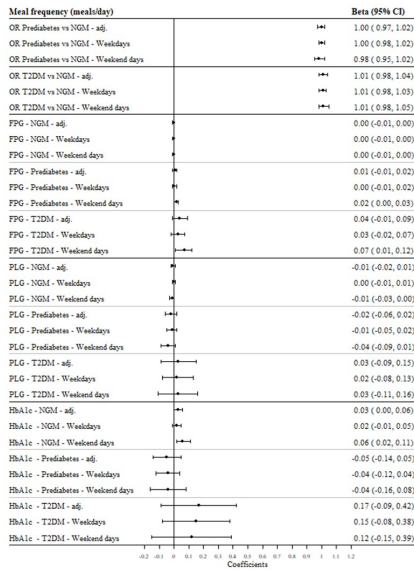

Comparison plot

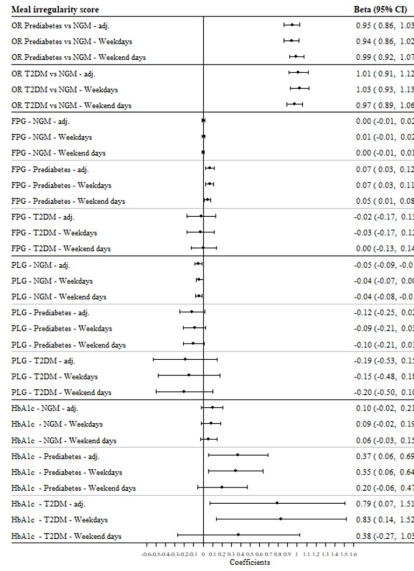

Comparison plot

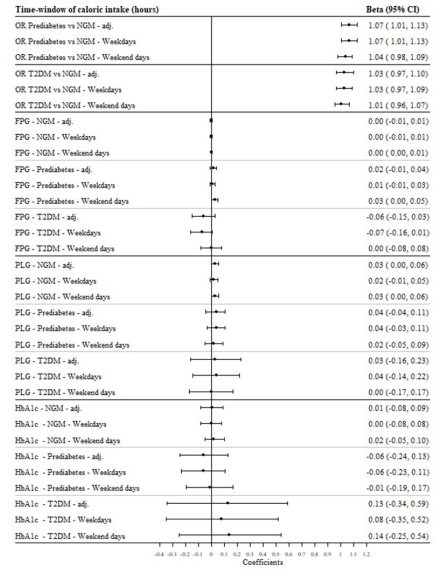

Comparison plot

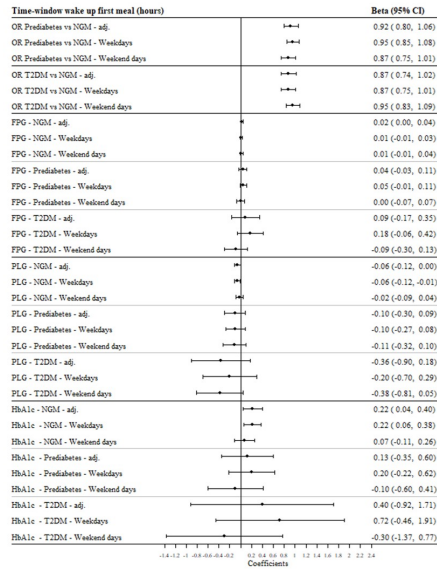

Comparison plot

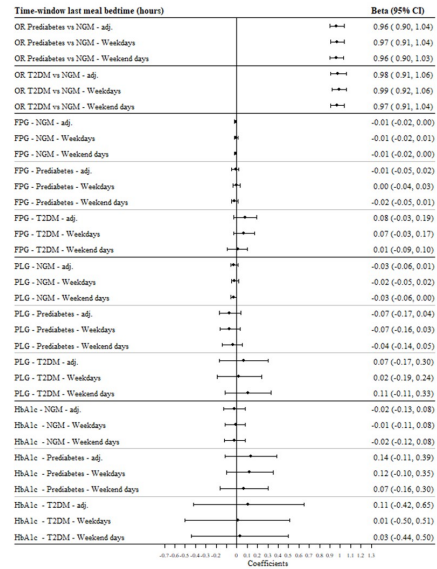

Supplement: Supplementary file 1 — Supplementary fig 1 Comparison plots illustrating the differences in adjusted continuous associations between weighted (week & weekend) chrono-nutrition variables and outcomes, as well as separate associations for chrono-nutrition variables based on week and weekend days alone. Abbreviations: CI, confidence interval; OR, odds ratio; NGM, normal glucose metabolism; adj., adjusted; T2DM, type 2 diabetes mellitus; FPG, fasting plasma glucose; PLG; 2-h post-load glucose; HbA1c, hemoglobin A1c. Odds ratios were estimated using multinomial logistic regression models, while associations with FPG, PLG, and HbA1c were analyzed using linear regression models. (PDF 895 kb) [file 394_2026_3964_MOESM1_ESM.pdf]

Comparison plot

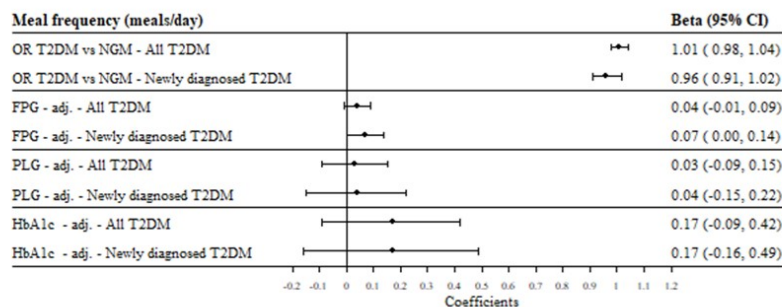

Comparison plot

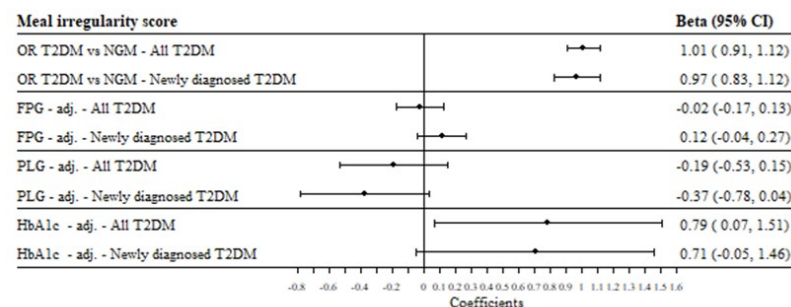

Comparison plot

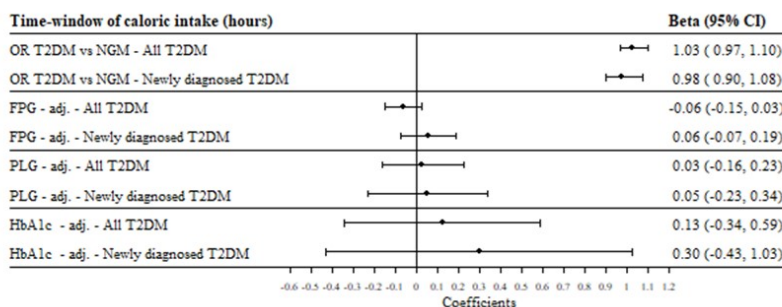

Comparison plot

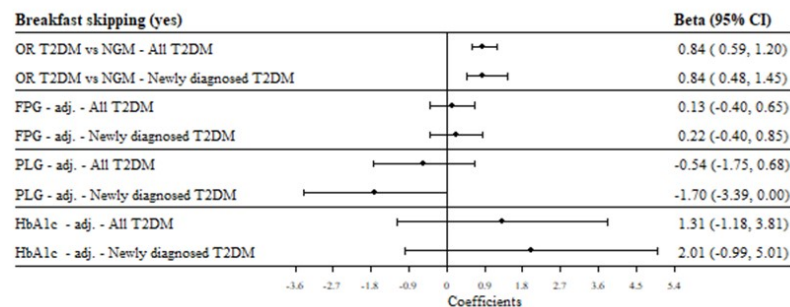

Supplement: Supplementary file 2 — Supplementary fig 2 Comparison plots illustrating the differences in adjusted continuous associations between chrono-nutrition and outcomes, for the whole T2DM group (as in main analyses) compared to only participants with newly diagnosed T2DM. Abbreviations: CI, confidence interval; OR, odds ratio; adj., adjusted; T2DM, type 2 diabetes mellitus; FPG, fasting plasma glucose; PLG; 2-h post-load glucose; HbA1c, hemoglobin A1c. Odds ratios were estimated using multinomial logistic regression models, while associations with FPG, PLG, and HbA1c were analyzed using linear regression models. (PDF 246 kb) [file 394_2026_3964_MOESM2_ESM.pdf]
