# Supplementary material for: Associations between chrono-nutrition and glucose metabolism across levels of glucose impairment: The Maastricht Study
Source: Eur J Nutr. 2026 Apr 21;65(4):119. doi: 10.1007/s00394-026-03964-2 (PMC13099856; doi:10.1007/s00394-026-03964-2)
Supplement: Supplementary file 3 — Supplementary file3 (DOCX 33 kb) [file 394_2026_3964_MOESM3_ESM.docx]

**Supplementary Table 1.** Confounder-adjusted associations of the wake-to-first meal and last meal-to-bed intervals with glucose metabolism outcomes within participants of The Maastricht Study

|  | **Q1^1,2^** | **Q2** | **Q3** | **Q4** | **Continuous,**  **Per one hour increase** |
| --- | --- | --- | --- | --- | --- |
|  | **OR (95% CI)** | **OR (95% CI)** | **OR (95% CI)** | **OR (95% CI)** | **OR^3^ (95% CI)** |
| **Multinominal logistic regression – Odds of having prediabetes/T2DM compared to NGM** | | | | | |
| **Wake-to-first meal interval** | | | |  |  |
| Prediabetes vs NGM | REF | 1.02 (0.74, 1.39) | 1.11 (0.82, 1.49) | 0.97 (0.71, 1.32) | 0.92 (0.80, 1.06) |
| T2DM vs NGM | REF | 0.88 (0.63, 1.23) | 1.04 (0.76, 1.43) | 0.83 (0.59, 1.17) | 0.87 (0.74, 1.02) |
|  |  |  |  |  |  |
| **Last meal-to-bed interval** | | | |  |  |
| Prediabetes vs NGM | REF | 0.90 (0.66, 1.22) | 1.03 (0.76, 1.38) | 0.86 (0.63, 1.17) | 0.96 (0.90, 1.04) |
| T2DM vs NGM | REF | 1.04 (0.74, 1.47) | 1.03 (0.74, 1.44) | 0.94 (0.67, 1.31) | 0.98 (0.91, 1.06) |
|  |  |  |  |  |  |
| **Linear regression – Stratified by glucose metabolism status^5^** | | | | | |
|  | **β (95% CI)** | **β (95% CI)** | **β (95% CI)** | **β (95% CI)** | **β^4^ (95% CI)** |
| Fasting plasma glucose concentration (mmol/L) | | | | | |
| **Wake-to-first meal interval** | | | |  |  |
| NGM | REF | -0.01 (-0.07, 0.04) | -0.02 (-0.07, 0.03) | 0.01 (-0.04, 0.07) | 0.02 (0.00, 0.04) |
| Prediabetes | REF | -0.10 (-0.26, 0.05) | -0.02 (-0.17, 0.12) | 0.00 (-0.15, 0.16) | 0.04 (-0.03, 0.11) |
| T2DM | REF | 0.29 (-0.23, 0.82) | -0.10 (-0.58, 0.38) | 0.16 (-0.37, 0.69) | 0.09 (-0.17, 0.35) |
|  |  |  |  |  |  |
| **Last meal-to-bed interval** | | | |  |  |
| NGM | REF | 0.02 (-0.03, 0.07) | -0.03 (-0.08, 0.02) | -0.02 (-0.08, 0.03) | -0.01 (-0.02, 0.00) |
| Prediabetes | REF | 0.04 (-0.11, 0.19) | 0.03 (-0.11, 0.17) | -0.07 (-0.22, 0.17) | -0.01 (-0.05, 0.02) |
| T2DM | REF | -0.38 (-0.90, 0.15) | 0.04 (-0.46, 0.55) | 0.31 (-0.19, 0.81) | 0.08 (-0.03, 0.19) |
| 2h post-load plasma glucose concentration (mmol/L) | | | | | |
| **Wake-to-first meal interval** | | | |  |  |
| NGM | REF | -0.09 (-0.24, 0.06) | -0.03 (-0.18, 0.11) | -0.13 (-0.29, 0.02) | -0.06 (-0.12, 0.00) |
| Prediabetes | REF | 0.01 (-0.44, 0.46) | 0.08 (-0.35, 0.50) | -0.20 (-0.66, 0.25) | -0.10 (-0.30, 0.09) |
| T2DM | REF | -0.55 (-1.67, 0.57) | 0.18 (-0.83, 1.18) | -0.62 (-1.73, 0.49) | -0.36 (-0.90, 0.18) |
|  |  |  |  |  |  |
| **Last meal-to-bed interval** | | | |  |  |
| NGM | REF | 0.03 (-0.12, 0.17) | -0.06 (-0.20, 0.09) | -0.07 (-0.22, 0.08) | -0.03 (-0.06, 0.01) |
| Prediabetes | REF | 0.06 (-0.38, 0.50) | 0.07 (-0.35, 0.49) | -0.18 (-0.63, 0.26) | -0.07 (-0.17, 0.04) |
| T2DM | REF | **-1.17 (-2.26, -0.07)** | -0.68 (-1.75, 0.39) | -0.11 (-1.18, 0.96) | 0.07 (-0.17, 0.30) |
| HbA1c concentration (mmol/mol) | | | | | |
| **Wake-to-first meal interval** | | | |  |  |
| NGM | REF | **0.55 (0.10, 1.00)** | 0.00 (-0.42, 0.43) | **0.55 (0.11, 0.99)** | **0.22 (0.04, 0.40)** |
| Prediabetes | REF | **1.15 (0.08, 2.22)** | 0.12 (-0.88, 1.13) | 0.85 (-0.24, 1.93) | 0.13 (-0.35, 0.60) |
| T2DM | REF | 0.73 (-1.88, 3.33) | -0.54 (-2.95, 1.86) | 0.56 (-2.07, 3.19) | 0.40 (-0.92, 1.71) |
|  |  |  |  |  |  |
| **Last meal-to-bed interval** | | | |  |  |
| NGM | REF | -0.06 (-0.48, 0.36) | -0.21 (-0.63, 0.21) | -0.05 (-0.49, 0.39) | -0.02 (-0.13, 0.08) |
| Prediabetes | REF | 0.15 (-0.90, 1.20) | 0.30 (-0.71, 1.31) | 0.38 (-0.68, 1.44) | 0.14 (-0.11, 0.39) |
| T2DM | REF | -0.96 (-3.62, 1.69) | 1.17 (-1.38, 3.71) | 0.28 (-2.22, 2.79) | 0.11 (-0.42, 0.65) |

*Abbreviations: Q, quartile; OR, odds ratio; CI, confidence interval; REF, reference; NGM, normal glucose metabolism; T2DM, type 2 diabetes mellitus; HbA1c, glycohemoglobin A1c.*

*^1^For the wake-to-first meal interval Q1 ranged from ≤ 0.11 hours, Q2 ranged from 0.14 – 0.48 hours, Q3 ranged from 0.50 – 0.64 hours, and Q4 ranged from ≥0.67 hours. For the last meal-to-bed interval Q1 ranged from ≤1.43 hours, Q2 ranged from 1.50 – 2.21 hours, Q3 ranged from 2.29 – 3.61 hours, and Q4 ranged from ≥3.62 hours.*

*^2^All associations were adjusted for age, sex, education level, BMI, physical activity levels, smoking status, energy intake, alcohol intake and dietary quality. Linear regression models stratified within participants with T2DM were additionally adjusted for the use of glucose lowering medication.*

*^3^The OR indicates the odds for having prediabetes/T2DM compared to the reference category of NGM if the wake-to-first meal interval or last meal-to-bed interval increases with one hour.*

*^4^The β indicates the average increase/decrease in the outcome variable if wake-to-first meal interval or last meal-to-bed interval increases with one hour.*

*^5^The number of participants included with a normal glucose metabolism was 1782 and 1823, whereas 459 and 469 participants had prediabetes, and 424 and 432 T2DM, for the wake-to-first meal interval and last meal-to-bed interval, respectively.*

**Supplementary Table 2.** Sensitivity analysis stratifying confounder-adjusted associations of meal irregularity and breakfast skipping with continuous glucose metabolism outcomes in The Maastricht Study participants with prediabetes into impaired fasting glucose and impaired glucose tolerance.

|  | **Q1^1,2^** | **Q2** | **Q3** | **Q4** | **Continuous,**  **Per one increase** |
| --- | --- | --- | --- | --- | --- |
|  | **β (95% CI)** | **β (95% CI)** | **β (95% CI)** | **β (95% CI)** | **β^3^ (95% CI)** |
| **Meal irregularity^4^** | | | | | |
| **Fasting plasma glucose concentration (mmol/L)** | | | |  |  |
| Prediabetes – Total^5^ | REF | 0.12 (-0.05, 0.29) | **0.25 (0.08, 0.43)** | **0.24 (0.05, 0.42)** | **0.07 (0.03, 0.12)** |
| Prediabetes – IFG | REF | 0.06 (-0.07, 0.19) | -0.01 (-0.14, 0.13) | -0.02 (-0.14, 0.09) | -0.01 (-0.04, 0.02) |
| Prediabetes – IGT | REF | 0.16 (-0.03, 0.36) | **0.29 (0.09, 0.50)** | 0.22 (-0.01, 0.46) | **0.07 (0.02, 0.13)** |
|  |  |  |  |  |  |
| **2h post-load plasma glucose concentration (mmol/L)** | | | |  |  |
| Prediabetes – Total | REF | -0.07 (-0.57, 0.44) | -0.30 (-0.83, 0.24) | -0.45 (-1.01, 0.10) | -0.12 (-0.25, 0.02) |
| Prediabetes – IFG | REF | -0.48 (-1.13, 0.18) | **-0.77 (-1.45, -0.09)** | -0.45 (-1.05, 0.15) | -0.08 (-0.23, 0.07) |
| Prediabetes – IGT | REF | -0.13 (-0.46, 0.20) | -0.09 (-0.44, 0.26) | 0.03 (-0.37, 0.42) | 0.02 (-0.07, 0.11) |
|  |  |  |  |  |  |
| **HbA1c concentration (mmol/mol)** | | | |  |  |
| Prediabetes – Total | REF | -0.23 (-1.43, 0.96) | 0.06 (-1.21, 1.32) | **1.46 (0.15, 2.77)** | **0.37 (0.06, 0.69)** |
| Prediabetes – IFG | REF | -1.14 (-3.53, 1.25) | -2.42 (-4.94, 0.09) | -0.48 (-2.70, 1.75) | -0.26 (-0.82, 0.31) |
| Prediabetes – IGT | REF | 0.00 (-1.42, 1.42) | 0.75 (-0.74, 2.25) | **1.81 (0.12, 3.50)** | **0.54 (0.16, 0.93)** |
|  |  |  |  |  |  |
| **Breakfast skipping, yes** | | | | | |
|  |  | **β^6^ (95% CI)** |  |  |  |
| **Fasting plasma glucose concentration (mmol/L)** | | | |  |  |
| Prediabetes – Total |  | 0.05 (-0.10, 0.21) |  |  |  |
| Prediabetes – IFG |  | 0.00 (-0.10, 0.09) |  |  |  |
| Prediabetes – IGT |  | 0.01 (-0.18, 0.20) |  |  |  |
|  |  |  |  |  |  |
| **2h post-load plasma glucose concentration (mmol/L)** | | | |  |  |
| Prediabetes – Total |  | **-0.50 (-0.96, -0.05)** |  |  |  |
| Prediabetes – IFG |  | -0.37 (-0.84, 0.11) |  |  |  |
| Prediabetes – IGT |  | -0.15 (-0.47, 0.17) |  |  |  |
|  |  |  |  |  |  |
| **HbA1c concentration (mmol/mol)** | | | |  |  |
| Prediabetes – Total |  | 0.85 (-0.19, 1.90) |  |  |  |
| Prediabetes – IFG |  | -0.99 (-2.69, 0.70) |  |  |  |
| Prediabetes – IGT |  | **1.61 (0.26, 2.96)** |  |  |  |

*Abbreviations: Q, quartile; CI, confidence interval; REF, reference; IFG, impaired fasting glucose; IGT, impaired glucose tolerance; HbA1c, glycohemoglobin A1c.*

*^1^All associations were adjusted for age, sex, education level, BMI, physical activity levels, smoking status, energy intake, alcohol intake and dietary quality.*

*^2^Q1 ranged from 0.00 – 1.21 average meal irregularity, Q2 ranged from 1.29 – 2.29 meal irregularity, Q3 ranged from 2.36 – 3.36 meal irregularity, and Q4 ranged from 3.43 – 6.00 meal irregularity.*

*^3^The β indicates the average increase/decrease in the outcome variable if the meal irregularity score increases with one.*

*^4^Higher scores for meal irregularity indicate a higher meal irregularity, with a score ranging from 0 (highly regular) to 6 (highly irregular).*

*^5^The total prediabetes group (N=604) consists of participants with impaired fasting glucose (N=176) and impaired glucose tolerance (N=428).*

*^6^The β indicates the average increase/decrease in the outcome variable if the breakfast is skipped at least once per week.*
